# Extended Peritoneal Dialysis and Renal Recovery in HIV-Infected Patients with Prolonged AKI: A Report of 2 Cases

**DOI:** 10.1155/2017/8620832

**Published:** 2017-07-06

**Authors:** Donlawat Saengpanit, Pongpratch Puapatanakul, Piyaporn Towannang, Talerngsak Kanjanabuch

**Affiliations:** ^1^Division of Nephrology, Department of Medicine, Faculty of Medicine, Chulalongkorn University, Bangkok, Thailand; ^2^CAPD Excellent Center, King Chulalongkorn Memorial Hospital, Bangkok, Thailand; ^3^Kidney and Metabolic Research Unit, Department of Medicine, Faculty of Medicine, Chulalongkorn University, Bangkok, Thailand

## Abstract

Peritoneal dialysis (PD) has recently been established as a treatment option for renal replacement therapy (RRT) in patients with acute kidney injury (AKI). Its efficacy in providing fluid and small solute removal has also been demonstrated in clinical trials and is equivalent to hemodialysis (HD). However, effect of RRT modality on renal recovery after AKI remains a controversy. Moreover, the setting of human immunodeficiency virus- (HIV-) infected patients with AKI requiring RRT makes the decision on RRT initiation and modality selection more complicated. The authors report here 2 cases of HIV-infected patients presenting with severe AKI requiring protracted course of acute RRT. PD had been performed uneventfully in both cases for 4–9 months before partial renal recovery occurred. Both patients eventually became dialysis independent but were left in chronic kidney disease (CKD) stage 4. These cases highlight the example of renal recovery even after a prolonged course of dialysis dependence. Thus, PD might be a suitable option for HIV patients with protracted AKI.

## 1. Introduction

Acute kidney injury (AKI) has been increasingly recognized in human immunodeficiency virus- (HIV-) infected patients and associated with poor outcomes [1]. The management of the HIV-infected patients with AKI requires meticulous attention to the control of fluid status, electrolytes, acid-base balance, and uremic toxin removal, in a similar manner as in those without HIV infection. Peritoneal dialysis (PD) is an overlooked modality of renal replacement therapy (RRT) for AKI as it is mainly considered in patients with end-stage renal disease (ESRD) [2]. However, acute PD remains a viable treatment option for selected cases of HIV-infected patients with AKI, especially in those who are hemodynamically unstable, having severe bleeding disorders, or when other modalities are unavailable [3]. PD has several advantages as an RRT in AKI patients including wide availability, ease of performance, nonvascular access placement, ability to remove large amounts of fluid in hemodynamically unstable patients, gradual but effective correction of acid-base and electrolyte imbalance, no need for anticoagulation, and high biocompatibility [3]. Above all, PD provides better preservation of residual kidney function (RKF) compared to hemodialysis (HD) in ESRD patients [4]. In those patients with potentially reversible etiology of AKI, treatment with PD may also have a higher likelihood of recovery of endogenous renal function [5].

We presented 2 cases of HIV-infected patients with severe AKI requiring acute RRT in which PD had been undertaken uneventfully. A few months later, recovery of renal function followed and PD was discontinued safely.

## 2. Case Report

### 2.1. Case  1

A 49-year-old Dutch male patient came to King Chulalongkorn Memorial Hospital (KCMH) with a complaint of low-grade fever and profuse sweating at night for 1 week. Three weeks earlier, he was diagnosed HIV infection when antiretroviral medications comprising of tenofovir, emtricitabine, and efavirenz were prescribed. He denied taking any over-the-counter drugs. The physical examination was unremarkable except for a body temperature of 38°C. He also had normotension (blood pressure 125/75 mmHg) without orthostatic hypotension or other signs of volume depletion. The chest X-ray showed miliary pulmonary nodules compatible with miliary tuberculosis which was later confirmed by positive polymerase-chain-reaction for* Mycobacterium tuberculosis* in his sputum. Disseminated tuberculosis was promptly diagnosed, and antituberculosis treatment (isoniazid, rifampicin, pyrazinamide, and ethambutol) was planned. However, he also had severe azotemia at admission (BUN 53.6 mmol/L, Cr 1,230 *µ*mol/L) in contrast to his baseline values from one month earlier (Cr 115 *µ*mol/L). At that time, there were no evidences of uremic symptoms or volume overload, and he still voided 500 mL of urine per day. Urinalysis revealed isosthenuria with bland urinary sediments (specific gravity 1.010, pH 5.0, albuminuria trace, glycose negative, WBC 0-1/hpf, and RBC 0-1/hpf). Renal ultrasonography demonstrated normal size and contour of both kidneys. Urine biomarker for renal tubular injury, neutrophil gelatinase-associated lipocalin (NGAL), was markedly elevated (7,891 ng/mL). Acute kidney injury was diagnosed and likely caused by nephrotoxic acute tubular necrosis (ATN) even though a renal biopsy had not been done. In the absence of other offending drugs or conditions, tenofovir was suspected to be a causal drug for ATN resulting in an adjustment of the antiretroviral regimen (abacavir, lamivudine, and raltegravir).

In the absence of uremic symptom or volume overload, PD was, nevertheless, initiated due to high level of nitrogen catabolites. The flexible double-cuffed PD catheter was inserted on day 4 of admission, and automated PD (Homechoice cycler®; Baxter) using a total dialysate (Dianeal®; Baxter) volume of 10 L (initial fill volume of 700 mL, 14 cycles, 20 hours) was promptly started on the same day of the catheter insertion. PD dose was gradually increased to achieve the total dialysate volume of 20 L per day in the next few days. The delivered dose of PD by total weekly Kt/V and total weekly creatinine clearance (CCr) were 3.23 and 97.84 L/week, respectively. After a week of automated PD, nitrogen catabolites decreased gradually (BUN 27.8 mmol/L, Cr 840 *µ*mol/L). At one month, his urine volume had increased to 1 L per day, but measured renal CCr was still at 4 mL/min/1.73 m^2^ which reflected inadequate recovery of renal function. He was discharged on day 31 of admission with continuation of automated PD at a total dialysate volume of 10 L per day. At follow-up visit, the patient showed gradually improvement in renal function and the dose of PD was tapered accordingly. Eventually, PD could be discontinued at 4 months after the onset of AKI. The patient attained stable serum Cr of 124 *µ*mol/L and measured CCr of 29 mL/min/1.73 m^2^ afterwards.

### 2.2. Case  2

A 58-year-old Thai female patient with hypertension, hyperlipidemia, and type 2 diabetes mellitus was infected with HIV 1.5 years ago. She had been taking antiretroviral drugs including tenofovir, emtricitabine, and boosted darunavir thereafter and achieved virological control after 6 months of therapy. Her CD4-positive T-lymphocyte count was 532/mm^3^ (40%). Her other medications were amlodipine 5 mg/day, enalapril 10 mg/day, fenofibrate 300 mg/day, and metformin 500 mg/day. She gradually developed anorexia, nausea, and fatigue over two weeks' duration. She also noticed a decrease in her daily urine volume and new-onset nocturia together with swelling in both of her legs particularly in the evening. She reported no fever, rash, or joint pain. She denied taking over-the-counter medication or nonsteroidal anti-inflammatory drugs. On examination, she was alert and had normal vital signs except for mild hypertension (body temperature 37.0°C, pulse rate 70/min, respiratory rate 16/min, and blood pressure 140/70 mmHg). She also had mildly pale conjunctivae and pitting edema in both of her legs. Laboratory tests showed severe azotemia (BUN 21.4 mmol/L, Cr 1,370 *µ*mol/L) compared to baselines labs 1 month earlier (Cr 124 *µ*mol/L). She also had hyponatremia, hypokalemia, metabolic acidosis, and elevated muscle enzyme (sodium 127 mEq/L, potassium 5.5 mEq/L, chloride 94 mEq/L, bicarbonate 10 mEq/L, and creatine phosphokinase 1,904 U/L; normal value 22–165 U/L). Urinalysis revealed isosthenuria, albuminuria, leukocyturia, and microhematuria without dysmorphic RBC (specific gravity 1.010, proteinuria 2+, glucose negative, WBC 3–5/hpf, and RBC 20–30/hpf). Renal ultrasonography demonstrated normal size but mildly increased parenchymal echogenicity of both kidneys without hydroureter or hydronephrosis. AKI was diagnosed. Differential diagnoses of AKI included tenofovir-induced ATN, HIV-associated nephropathy/immune complex glomerulonephritis, and rhabdomyolysis.

RRT was initiated soon after admission due to uremia and volume overload. After successful insertion of flexible double-cuff PD catheter, automated PD (Homechoice cycler; Baxter) using total dialysate (Dianeal; Baxter) volume of 10 L was started (fill volume of 2 L, five cycles, therapy time 12 hours) on the first day of admission resulting in adequate control of fluid, electrolytes, and acid-base balance. The doses of PD by total weekly Kt/V and total weekly CCr were 3.63 and 91.94 L/week, respectively. Renal biopsy was later performed revealing evidence of acute granulomatous interstitial nephritis (AIN) and ATN without evidence of glomerular or vascular injury. Antiretroviral drugs-induced ATN/AIN was diagnosed. The attending physician then switched antiretroviral regimen to stavudine/lamivudine/boosted darunavir regimen. During fourth week of admission, her urine volume had increased to 0.8–1.0 L per day but the measured renal CCr was still low (6.62 mL/min/1.73 m^2^). She was discharged from the hospital anyway and was prescribed to continue automated PD during night time at home (night intermittent PD; NIPD) at a similar dose (fill volume of 2 L, five cycles, therapy time 12 hours). Eventually, PD was successfully discontinued 9 months after the onset of AKI in August 2014. At that time, her serum Cr was 159 *µ*mol/L, and measured renal CCr was stable at 17.3 mL/min/1.73 m^2^ with daily urine volume of 2,480 mL. Afterwards, she remained in chronic kidney disease (CKD) stage 4 with stable renal function for another whole year.

## 3. Discussion

AKI has been demonstrated to occur at a 2 to 3 times higher incidence in hospitalized HIV-infected patients compared to those without HIV infection and is associated with poor long-term outcomes, including increased risk of cardiovascular events, ESRD, and mortality [1]. The common causes of AKI in HIV-infected patients are volume depletion, septicemia, and nephrotoxic medications [6]. In cases where RRT is warranted, acute PD has been established as a viable option for selected patients, particularly those who are hemodynamically unstable and have severe coagulation defects, or when other modalities are not readily available [2]. Moreover, performing PD in HIV-infected patients reduces exposure of healthcare workers to contaminated blood and needle, putting them at a lower risk of acquiring the infection. As for patients' outcomes, PD has been shown to provide better preservation of residual kidney function (RKF) in long-term dialysis patients compared to HD [4]. In AKI setting, the outcomes of patients including survival and metabolic control were comparable between daily intermittent HD and PD using high volume prescription (36–44 L of dialysate per day) [5].

Renal recovery after AKI in HIV-infected patients has not been well-described. In a large cohort of 489 hospitalized HIV-infected patients, in which 18% developed AKI, renal recovery occurred in 67.2%, and rate of recovery decreased with increasing severity of AKI according to “Risk Injury Failure Loss of kidney function End-stage kidney disease” (RIFLE) criteria (Risk, 85.2%; Injury 61.9%; Failure, 43.8%) [7]. Time to renal recovery of AKI in HIV-infected is also rarely described. Generally, renal recovery in AKI, especially in cases of ATN, usually occurs within an average of 1–3 weeks. However, on a rare occasion, it may take up to several months for kidney to recover, mostly dependent on severity and duration of the insulting causes of ATN [8]. There is conflicting evidence whether PD helps renal recovery in a patient with AKI. One randomised controlled trial in Brazil comparing high volume PD and daily intermittent HD in AKI patients showed similar rate of recovery in both groups (28% versus 26%; *p* = 0.84) but patients undergoing PD had shorter time to renal recovery (7.2 ± 2.6 days versus 10.6 ± 4.7 days; *p* = 0.04) [5]. On the other hand, in another randomised controlled trial comparing high volume PD and extended HD (duration 6–8 hours per session, 6 times per week) in 143 critically ill patients with ATN, rate of renal recovery and time to recovery were similar in two groups [9]. Whether PD has better effect on renal recovery compared to HD remained controversial.

Many possible mechanisms may account for the better preservation of RKF in PD and may involve in renal recovery after an episode of AKI. For example, abrupt fluctuation in volume and osmotic load are fewer in PD patients, so their hemodynamic status during dialysis is more stable than HD. This may be associated with more stable glomerular capillary pressure and more constant glomerular filtration. An episode of renal ischemia caused by rapid fluctuation of osmolality and contraction of the circulatory volume is more frequent during HD than PD. Mild overhydration of some patients on PD may contribute to better RKF preservation. The membrane used in hemodialysers is less biocompatible than the peritoneal membrane, leading to rapid loss of RKF caused by repeated exposure to inflammatory mediators such as IL-1 that generated by the extracorporeal circulation [10].

Our patients presented with severe nonoliguric AKI caused by ATN/AIN both of which underwent PD as an RRT modality. Despite early discontinuation of the offending drugs that were suspected to be the cause of AKI, together with the use of PD, both patients showed rather slow recovery of renal function and remained dialysis-dependent after being discharged from the hospital for a few months. Since there were no repeated hemodynamic collapse, infective complications, or other episodes of AKI, the delayed renal recovery in these patients was likely contributed by severity of the AKI itself. In this scenario of AKI with late renal recovery requiring extended RRT, there is no data whether HD or PD will have superior benefit. Technically, PD put patients at a lower risk of hemodynamic instability as the fluid and metabolites removal occurs slowly, and with no need for using hemodialyser membrane as in HD, performing PD has less inflammation due to bioincompatibility, both of which may promote recovery of renal function. Both of our cases finally attained partial recovery of renal function and became dialysis independent. However, some kidney damage remained, putting them in CKD status. Finally, choosing PD for acute RRT may help decrease financial burden of healthcare systems especially in cases of prolonged AKI as in our cases.

## 4. Conclusion

AKI is common in HIV-infected patients and associated with poor outcomes. Performing PD in HIV-infected patients with AKI provides not only similar efficacy in fluid and metabolic control as other extracorporeal treatments, but also potentially superior ability to increase the likelihood of renal recovery, particularly in those with prolonged course requiring extended RRT. Moreover, PD in HIV-infected patients lowers exposure of healthcare worker to contaminated blood and decreases financial burden of healthcare systems especially in cases of delayed recovery. In summary, PD possesses several advantages and is a suitable RRT option for HIV-infected patients presenting with AKI.
